# Isaridin E Protects against Sepsis by Inhibiting Von Willebrand Factor-Induced Endothelial Hyperpermeability and Platelet–Endothelium Interaction

**DOI:** 10.3390/md22060283

**Published:** 2024-06-16

**Authors:** Yao-Sheng Liu, Wen-Liang Chen, Yu-Wei Zeng, Zhi-Hong Li, Hao-Lin Zheng, Ni Pan, Li-Yan Zhao, Shu Wang, Sen-Hua Chen, Ming-Hua Jiang, Chen-Chen Jin, Yu-Chen Mi, Zhao-Hui Cai, Xin-Zhe Fang, Yong-Jun Liu, Lan Liu, Guan-Lei Wang

**Affiliations:** 1 Department of Pharmacology, Zhongshan School of Medicine, Sun Yat-sen University, Guangzhou 510080, China; liuysh55@mail2.sysu.edu.cn (Y.-S.L.); zengyw25@mail2.sysu.edu.cn (Y.-W.Z.); l1352272780@163.com (Z.-H.L.); zhaoyan0809@hotmail.com (L.-Y.Z.); medpharm_ws@163.com (S.W.); 18826478932@163.com (C.-C.J.); miych@mail2.sysu.edu.cn (Y.-C.M.); caizhh28@mail2.sysu.edu.cn (Z.-H.C.); fangxzh3@mail2.sysu.edu.cn (X.-Z.F.); 2 Scientific Research Center, the Medical Interdisciplinary Science Research Center of Western Guangdong, College of Women and Children, the Second Affiliated Hospital of Guangdong Medical University, Zhanjiang 524023, China; febright@126.com; 3 Division of Biosciences, University College London, London WC1E 6BT, UK; leo.zheng.22@ucl.ac.uk; 4 Department of Pharmacy, The Second Clinical College, Guangzhou Medical University, Guangzhou 510261, China; pann@mail2.sysu.edu.cn; 5 School of Marine Sciences, Sun Yat-sen University, Guangzhou 510006, China; chensenh@mail.sysu.edu.cn (S.-H.C.); jiangmh23@mail2.sysu.edu.cn (M.-H.J.); 6 Southern Marine Sciences and Engineering Guangdong Laboratory (Zhuhai), Zhuhai 519000, China; 7 Guangdong Provincial Clinical Research Center of Critical Care Medicine, Guangzhou 510080, China; 8 Department of Critical Care Medicine, The First Affiliated Hospital of Sun Yat-sen University, Guangzhou 510080, China

**Keywords:** sepsis, endothelial hyperpermeability, von Willebrand factor, isaridin E, acute lung injury

## Abstract

Endothelial hyperpermeability is pivotal in sepsis-associated multi-organ dysfunction. Increased von Willebrand factor (vWF) plasma levels, stemming from activated platelets and endothelium injury during sepsis, can bind to integrin αvβ3, exacerbating endothelial permeability. Hence, targeting this pathway presents a potential therapeutic avenue for sepsis. Recently, we identified isaridin E (ISE), a marine-derived fungal cyclohexadepsipeptide, as a promising antiplatelet and antithrombotic agent with a low bleeding risk. ISE’s influence on septic mortality and sepsis-induced lung injury in a mouse model of sepsis, induced by caecal ligation and puncture, is investigated in this study. ISE dose-dependently improved survival rates, mitigating lung injury, thrombocytopenia, pulmonary endothelial permeability, and vascular inflammation in the mouse model. ISE markedly curtailed vWF release from activated platelets in septic mice by suppressing vesicle-associated membrane protein 8 and soluble N-ethylmaleide-sensitive factor attachment protein 23 overexpression. Moreover, ISE inhibited healthy human platelet adhesion to cultured lipopolysaccharide (LPS)-stimulated human umbilical vein endothelial cells (HUVECs), thereby significantly decreasing vWF secretion and endothelial hyperpermeability. Using cilengitide, a selective integrin αvβ3 inhibitor, it was found that ISE can improve endothelial hyperpermeability by inhibiting vWF binding to αvβ3. Activation of the integrin αvβ3-FAK/Src pathway likely underlies vWF-induced endothelial dysfunction in sepsis. In conclusion, ISE protects against sepsis by inhibiting endothelial hyperpermeability and platelet-endothelium interactions.

## 1. Introduction

Sepsis, a life-threatening disease caused by an excessive response to infection, manifests with multiple organ dysfunctions, notably acute lung injury [1]. Globally, there were approximately 48.9 million reported sepsis cases in 2017, contributing to 11.0 million deaths and constituting 19.7% of total deaths [2]. Despite its prevalence and high mortality, effective treatments remain elusive, underscoring the urgent need to develop intervention targets and therapeutic agents.

The progression of multiple organ failure is correlated with sepsis mortality. Endothelial hyperpermeability has emerged as a major cause of this phenomenon over the last two decades [3]. Early in septic infection, both exogenous pathogen-associated and endogenous damage-associated molecular patterns activate the vascular endothelium, leading to structural and/or functional damage to endothelial cells (ECs). This disruption causes decreased endothelial barrier integrity, interstitial oedema, and tissue hypoperfusion, which lead to sepsis-associated multi-organ failure. Several functional proteins, such as syndecan-1 and VE-cadherin, have been proposed as diagnostic and prognostic markers for sepsis due to their roles in regulating endothelial hyperpermeability. Strategies targeting endothelial hyperpermeability were able to attenuate sepsis in experimental models by limiting vascular leakage and inflammation and reversing maladaptive EC responses to infection during sepsis [3]. A deeper understanding of the molecular mechanisms regulating endothelial hyperpermeability will help to identify novel targets for sepsis treatment.

von Willebrand factor (vWF), crucial in hemostasis and thrombosis, bridges activated platelets and ECs and serves as a marker of endothelial damage and thrombotic risk in sepsis and other serious infectious diseases, particularly COVID-19 [4]. vWF is synthesized and stored exclusively in ECs and megakaryocytes in basal levels, and which is routinely used to stain or identify vascular ECs in tissue sections. The increased vWF is observed during endothelial damage [5]. Upon activation of platelets and/or injury of endothelium, there is a rapid, substantial local release of ultra-large vWF (ULvWF) at the site of injury. ADAMTS-13 (a disintegrin and metalloproteinase with a thrombospondin type 1 motif, member 13) is responsible for cleaving ULvWF into smaller pieces, thereby reducing its plasma levels and activity. Elevated vWF/ADAMTS-13 ratios have been observed in sepsis and COVID-19 and are positively correlated with high ICU mortality [6,7]. Recently, the vWF/ADAMTS-13 axis has emerged as a potential therapeutic target for both sepsis and COVID-19. The integrin αvβ3 receptor in ECs facilitates the binding of acutely secreted vWF to ECs under fluid shear stress through the interaction between vWF and integrin αvβ3 [8]. Additionally, integrin αvβ3 has been implicated in sepsis-related endothelial injury in in vitro EC models of SARS-CoV-2 infection as well as infections with *Staphylococcus aureus* and *Escherichia coli* [9,10]. However, the pathological significance of vWF-integrin αvβ3 interactions in sepsis-associated endothelial hyperpermeability and the underlying molecular mechanism remain unclear.

Platelet activation serving a catalytic spot for thrombin formation is associated with sepsis severity. Platelets that interplay with endothelium are essential for maintaining endothelium integrity in resting [11]. Platelet hyperactivity occurring during sepsis causes disruption or dysfunction of the endothelium, leading to intravascular coagulation, which is the critical stage of multiple organ failure [12]. Our previous investigation revealed that isaridin E (ISE), a cyclodepsipeptide derived from the marine fungus *Amphichorda felina* SYSU-MS7908 sourced from the South China Sea, displayed antiplatelet and antithrombotic effects in both in vitro and in vivo thrombosis formation models induced by adenosine diphosphate (ADP) and FeCl_3_, respectively [13]. Notably, we observed that the bleeding risk of ISE in vivo was much lower compared to clopidogrel, and ISE did not exhibit cytotoxicity to mouse platelets at doses below 400 μM [13]. These findings motivated us to further explore its efficacy in a thrombotic disease animal model, specifically sepsis, wherein platelet hyperactivity is critical to its pathogenesis.

In this study, our objective was to investigate the therapeutic outcomes of ISE on sepsis and reveal the relevant underlying molecular mechanisms.

## 2. Results

### 2.1. ISE Increased the Survival Rate While Alleviating Acute Lung Injury and Systemic Inflammation in CLP-Induced Septic Mice

**Figure 1:**
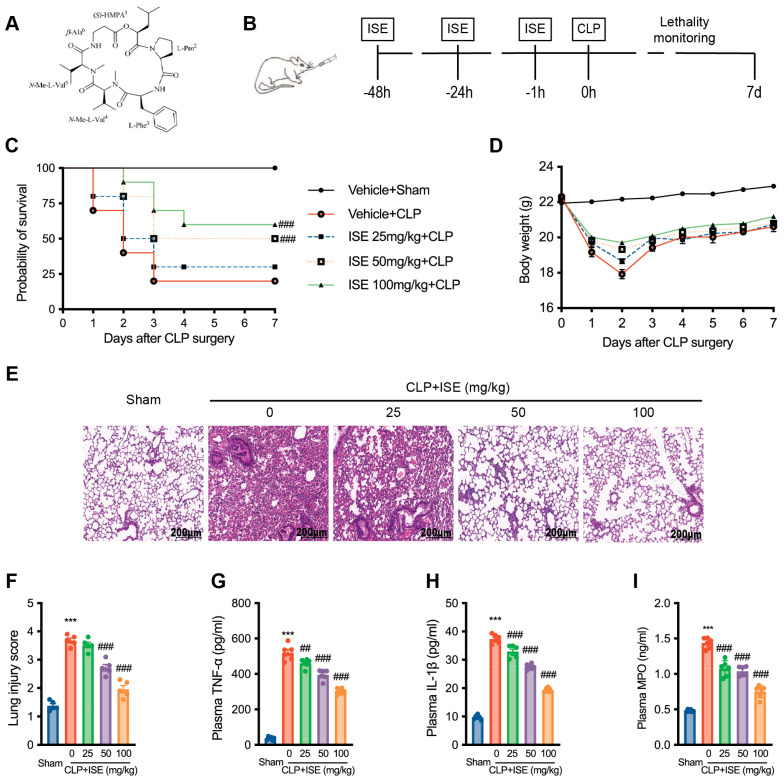
Effects of ISE on survival, lung injury, and systemic inflammation in septic mice. (**A**) Chemical structure of ISE. (**B**) Schematic overview of the experimental procedure. Mice were pretreated with multiple intragastric administrations of ISE (25, 50, or 100 mg/kg) or vehicle at 48 h, 24 h, and 1 h before the CLP operation. (**C**) Mice survival was monitored every 6 h for 7 consecutive days. Kaplan–Meier survival curves were used to analyze the data (*n* = 10 mice per group). The significance was evaluated by the log-rank (Mantel–Cox) test. (**D**) Body weights were measured and plotted every day for 7 days after the CLP surgery. (*n* = 2–10 mice per group). (**E**) Representative histological images of the lungs of control (vehicle-injected) and ISE-treated mice via H&E staining. Scale bar = 200 μm. (**F**) Lung tissue damage scores were evaluated in H&E-stained sections (five randomly chosen sections per mice, *n* = 5 mice per group). (**G**–**I**) Plasma inflammatory cytokine levels of TNF-α, IL-1β, and MPO after 12 h CLP surgery were determined using ELISA (*n* = 6 mice per group). *** *p* < 0.001 *vs*. the sham group; ^##^ *p* < 0.01; ^###^ *p* < 0.001 *vs*. the vehicle + CLP group.

We examined the impact of ISE on sepsis-induced mortality. Mouse survival was monitored for 7 days post-CLP with or without ISE pretreatment (25, 50, and 100 mg/kg) (Figure 1B). Mice with CLP-induced sepsis had a 7-day survival rate of 20%, which increased to 30%, 50%, and 60% with pretreatment of 25, 50, and 100 mg/kg ISE, respectively (*n* = 10 per group, Figure 1C). Body weight is a crucial indicator of sepsis severity. Mice subjected to CLP-induced sepsis, with or without ISE pretreatment, experienced initial weight loss for the first three days, followed by weight recovery. Notably, 50 or 100 mg/kg ISE significantly alleviated sepsis-induced weight loss (Figure 1D).

We assessed the effects of ISE on lung injury in septic mice. Hematoxylin and eosin-stained sections of lung tissues revealed significant interalveolar septal thickening, interstitial hemorrhage, and microvascular thrombus formation in CLP-induced septic mice, indicative of CLP-induced acute lung injury (Figure 1E, F, ***p < 0.001). ISE pretreatment (50 and 100 mg/kg) significantly ameliorated sepsis-induced lung injury (Figure 1E) and reduced lung injury scores (*n* = 5 per group, ^###^*p* < 0.001; Figure 1F). Additionally, in CLP-induced septic mice, we observed a significant increase in the levels of TNF-α, IL-1β, and MPO in plasma, which were significantly reduced by ISE pretreatment. The levels of TNF-α, IL-1β, and MPO in CLP-induced septic mice were 520.3 ± 17.8 pg/mL, 37.3 ± 0.6 pg/mL, and 1.4 ± 0.03 ng/mL, respectively. ISE pretreatment at doses of 25, 50, and 100 mg/kg reduced the plasma levels of TNF-α by 10.9 ± 4.1%, 23.6 ± 3.5%, and 41.3 ± 2.5%, IL-1β by 12.0 ± 0.9%, 26.1 ± 0.5%, and 48.0 ± 1.6%, and MPO by 24.5 ± 4.6%, 27.5 ± 2.5%, and 47.3 ± 2.7%, respectively. (^##^ *p* < 0.01, ^###^ *p* < 0.001 *vs*. the vehicle + CLP group, Figure 1G–I).

### 2.2. ISE Improved Pulmonary Vascular Permeability

**Figure 2:**
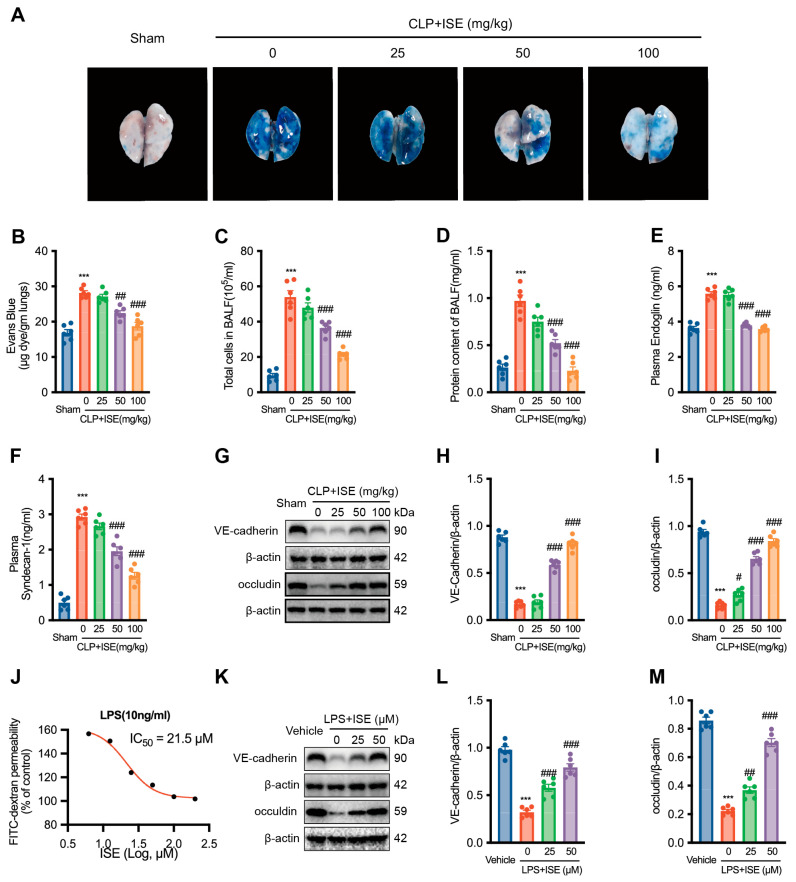
ISE significantly improved pulmonary endothelial permeability. (**A**) Representative images of lungs from mice treated via the tail vein with Evans blue staining. (**B**) Evans blue dye contents in the lungs. (**C**,**D**) The levels of cell count and protein content in bronchoalveolar lavage fluid. (**E**,**F**) The plasma levels of endoglin and syndecan-1 were determined by ELISA. (**G**–**I**) Western blot results of VE-cadherin and occludin expression in mouse lung tissue. (**J**) The IC_50_ value of ISE for the increased endothelial permeability induced by 10 ng/mL LPS is 21.5 μM. (**K**–**M**) Western blot results of VE-cadherin and occludin expression in LPS-stimulated HUVECs. *n* = 6 independent experiments. *** *p* < 0.001 *vs*. the sham group (animal) or the control group (cell); ^#^ *p* < 0.05; ^##^ *p* < 0.01, ^###^ *p* < 0.001 *vs*. the vehicle + CLP group (animal) or LPS group (cell).

Evans blue staining indicated significantly elevated pulmonary endothelial permeability in septic mice 12 h post-CLP (*n* = 6 per group, Figure 2A,B). As shown in Figure 2B, ISE at doses of 50 mg/kg and 100 mg/kg significantly decreased the CLP-induced maximum Evans blue penetration to 79.8 ± 2.4% and 66.7 ± 4.3% of the vehicle + CLP group. Moreover, CLP increased the levels of bronchoalveolar lavage fluid proteins and cells, which were reduced by ISE pretreatment (*n* = 6 per group, Figure 2C,D). These findings demonstrate that ISE may guard against sepsis-induced loss of endothelial barrier integrity.

CLP caused significant increases in plasma endoglin and syndecan-1 (*n* = 6 per group, Figure 2E, F) along with decreased tissue VE-cadherin and occludin levels (*n* = 6 per group, Figure 2G–I) in septic mice, which were ameliorated by ISE pretreatment. This suggests that ISE may have direct protective effects on lung vascular ECs, rendering the lungs less susceptible to sepsis-induced endothelial dysfunction. Furthermore, the effects of ISE on LPS-stimulated HUVECs were examined using an FITC-dextran assay. Incubation of HUVECs with 10 ng/mL LPS for 6 h substantially increased FITC-dextran permeability, while ISE significantly decreased this permeability with an IC_50_ of 21.5 μM (*n* = 6 per group, Figure 2J). Next, we chose the ISE concentration of 25 μM and 50μM for the subsequent intervention experiments. Consistently, ISE pretreatment significantly reduced the LPS-induced increase in VE-cadherin and occludin protein expression in HUVECs (*n* = 6 per group, Figure 2K–M). Overall, these results suggest that ISE acts directly on vascular ECs, thereby mitigating increased pulmonary vascular permeability and lung injury induced by CLP sepsis.

### 2.3. ISE Suppressed Sepsis-Associated vWF Release

**Figure 3:**
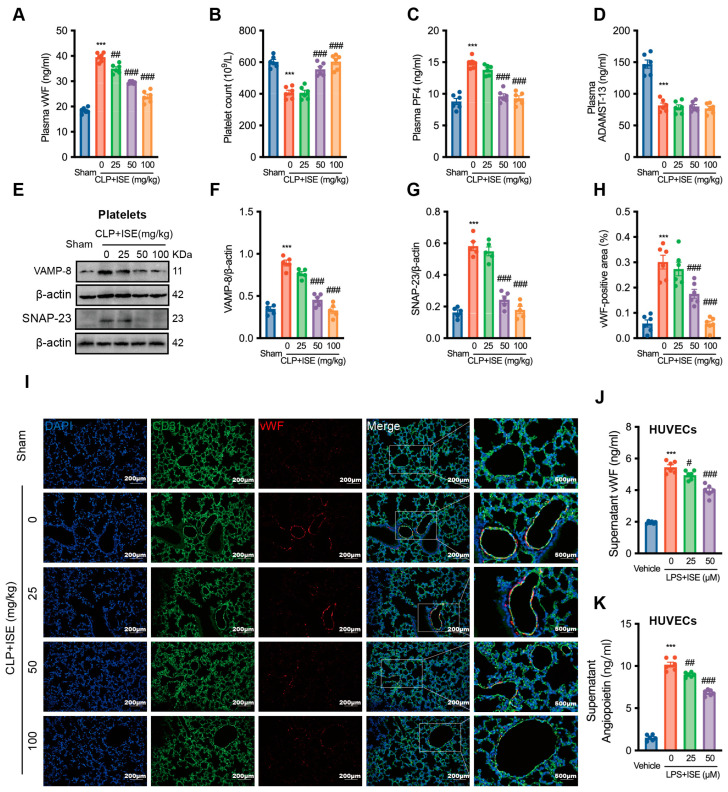
ISE inhibited vWF release under septic conditions. (**A**) The plasma levels of vWF were determined by ELISA. *n* = 6 mice per group. (**B**) Platelet count in mice. *n* = 6 mice per group. (**C**,**D**) The ELISA results of serum PF4 and ADAMST-13 in mice. *n* = 6 mice per group. (**E**–**G**) Western blot results of VAMP-8 and SNAP-23 expression in mouse platelets. *n* = 5 independent experiments. (**H**,**I**) Immunofluorescence staining results for vWF (red) expression in lung tissues and CD31 staining (green) were used as an endothelium marker. Scale bar = 200 μm and 500 μm, respectively. *n* = 6 mice per group. (**J**,**K**) VWF and angiopoietin 2 in the supernatant of HUVECs. *n* = 6 independent experiments. *** *p* < 0.001 *vs*. the sham group (animal) or the control group (cell); ^#^ *p* < 0.05; ^##^ *p* < 0.01, ^###^ *p* < 0.001 *vs*. the vehicle + CLP group (animal) or LPS group (cell).

ISE significantly inhibited ATP release from activated platelets [8]. Activated platelets and injured endothelium are the primary sources of vWF, and vWF in injured endothelium plays an important role in the development of pulmonary embolism, platelet consumption, and multiple organ dysfunction during severe infectious diseases, such as sepsis and severe COVID-19 infection [14,15]. At doses of 25, 50, and 100 mg/kg, ISE reduced the plasma vWF level by 11.4 ± 2.5%, 25.3 ± 1.6%, and 39.4 ± 2.4%, respectively, in comparison to the corresponding vehicle + CLP group (*n* = 6 per group, Figure 3A). Additionally, CLP-induced sepsis also decreased the platelet count from (602.6 ± 14.7) × 10^9^/L to (408.3 ± 15.3) × 10^9^/L, which was improved by ISE pretreatment (*n* = 6 per group, Figure 3B). Specifically, ISE (100 mg/kg) restored the decreased platelet count in CLP-septic mice to a level comparable to that in the sham group.

Under diseased conditions, activated platelets and injured ECs are major sources of high levels of circulating vWF. We therefore examined whether ISE regulates the release of vWF from platelets and ECs during sepsis. ISE pretreatment significantly reduced elevated PF4 levels (*n* = 6 per group, Figure 3C), but did not affect the decreased ADAMST-13 levels in the plasma of CLP-septic mice (*n* = 6 per group, Figure 3D). Soluble N-ethylmaleide-sensitive factor attachment protein 23 (SNAP-23) and vesicle-associated membrane protein 8 (VAMP-8) are responsible for granule secretion during platelet activation. ISE pretreatment diminished enhanced SNAP-23 and VAMP-8 protein expression in the platelets of CLP-induced sepsis mice (*n* = 5 independent experiments, Figure 3E–G). Injured ECs are another important source of vWF in plasma. For a more intuitive observation, we used immunofluorescence to observe the effect of ISE on vWF derived from sepsis lung ECs. As shown in Figure 3H, I, pretreatment with 50 mg/kg and 100 mg/kg of ISE significantly reduced vWF expression in the pulmonary vascular endothelium of CLP-induced sepsis mice (vehicle + CLP group, 0.30 ± 0.03% *vs*. 50 mg/kg + ISE CLP, 0.18 ± 0.02%, ^###^ *p* < 0.001; *vs*. 100 mg/kg ISE + CLP, 0.06 ± 0.01%, ^###^ *p* < 0.001). The results in Figure 3H, I clearly show that ISE specifically reduced vWF synthesized in injured ECs under the CLP–sepsis condition. Moreover, preincubation with 25 μM and 50 μM ISE significantly suppressed LPS-induced vWF secretion in HUVECs to 91.9 ± 2.7% and 72.8 ± 2.0% of cells treated with vehicle + LPS. (*n* = 6 independent experiments, Figure 3J). Angiopoietin 2 serves as a marker of Weibel–Palade bodies during EC secretion, and its LPS-induced increase was inhibited by ISE pretreatment of HUVECs (*n* = 6 independent experiments, Figure 3K). Overall, these results suggest that ISE may reduce locally high accumulation of vWF by decreasing its secretion from activated platelets and injured ECs during sepsis.

### 2.4. ISE Inhibited LPS-Induced Platelet–EC Interaction

**Figure 4:**
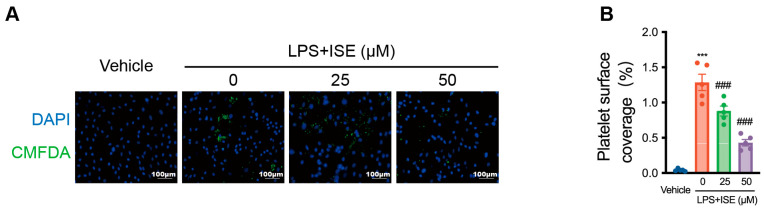
ISE inhibited LPS-induced platelet–EC interaction. (**A**) The representative fluorescent images of co-incubation experiments. Cultured HUVECs were pretreated with vehicle, LPS, or LPS + ISE, and the adhesion rate of isolated platelets (CellTracker™ Green CMFDA Dye, green) to HUVECs (DAPI, blue) was analyzed. Scale bar = 100 μm. (**B**) Quantitative analysis of platelet adhesion to HUVECs. *n* = 5 independent experiments. *** *p* < 0.001 vs. the control group; ^###^ *p* < 0.001 *vs*. the LPS group.

ISE pretreatment significantly decreased the increase in vWF caused by sepsis (Figure 3). Therefore, whether ISE affected vWF-mediated platelet–EC interactions was investigated. Fluorescence analysis showed that LPS stimulation of HUVECs increased the adhesion of platelets to HUVECs (*n* = 5 independent experiments, Figure 4A,B). This effect was inhibited by ISE in a concentration-dependent manner (LPS group, 1.3 ± 0.1% *vs*. LPS + 25 μM ISE group, 0.9 ± 0.1%, ^###^ *p* < 0.001, LPS group 1.3 ± 0.1% *vs*. LPS + 50 μM ISE group 0.4 ± 0.1%, ^###^ *p* < 0.001, Figure 4).

### 2.5. Effects of ISE on the Integrin αvβ3-FAK/Src Signalling Pathway

**Figure 5:**
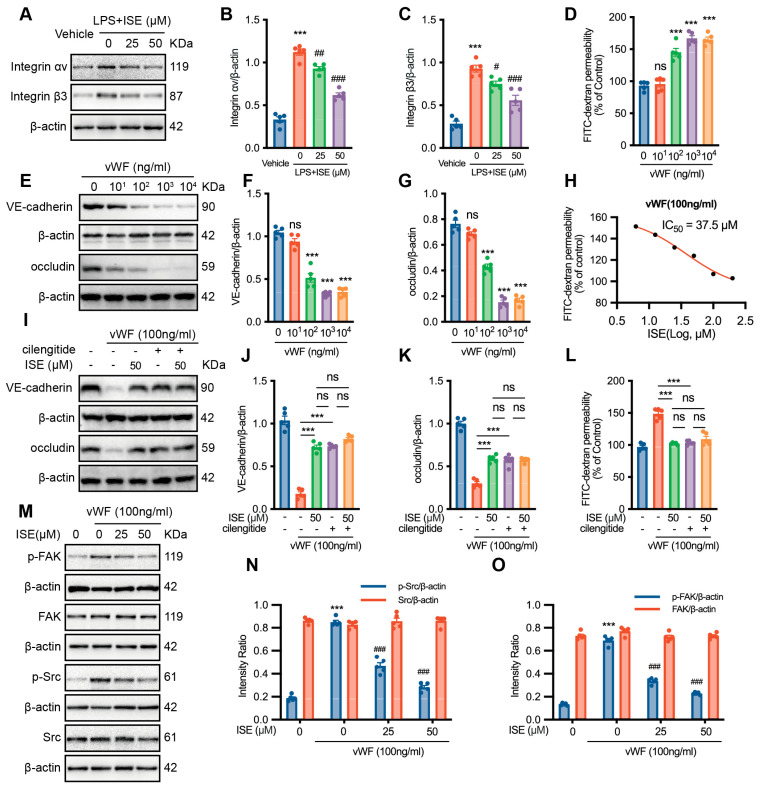
ISE inhibited the αVβ3-FAK/Src signaling pathway in vWF-stimulated HUVECs. (**A**–**C**) Western blotting analysis of integrin β3 and integrin αv expressions in HUVECs pretreated with LPS and different concentrations of ISE. *n* = 5 independent experiments. *** *p* < 0.001 *vs*. the vehicle group; ^#^ *p* < 0.05, ^##^ *p* < 0.01, ^###^ *p* < 0.001 *vs*. the LPS group. (**D**) Evaluation of endothelial permeability in vWF-stimulated HUVECs using the FITC-dextran assay. ns *p* > 0.05, *** *p* < 0.001 *vs.* the vehicle group. (**E**–**G**) Western blotting analysis of VE-cadherin and occludin expression in HUVECs pre-incubated with vehicle or different concentration of vWF. ns *p* > 0.05, *** *p* < 0.001 *vs.* the vehicle group. (**H**) The IC_50_ value of ISE for the increased endothelial permeability induced by 100 ng/mL vWF is 37.5 μM. (**I**–**K**) VE-cadherin and occludin expression in vWF-stimulated HUVECs following pretreatment with ISE or cilengitide alone or ISE + cilengitide. ns *p* > 0.05, *** *p* < 0.001. (**L**) Evaluation of endothelial permeability using the FITC-dextran assay. ns *p* > 0.05, *** *p* < 0.001. (**M**–**O**) Western blotting analysis of total and phosphorylated FAK (Y397) and Src (Try416) expressions in vWF-stimulated HUVECs pretreated with different concentrations of ISE. *n* = 5 independent experiments. *** *p* < 0.001 *vs*. the vehicle group; ^###^ *p* < 0.001 *vs*. the vWF group.

Integrin αvβ3, an adhesion receptor for vWF, has emerged as a novel therapeutic target for treating sepsis and COVID-19 by lowering enhanced endothelial permeability [16]. Cilengitide, a selective αvβ3/αvβ5 integrin antagonist [17], was employed to assess αvβ3 protein expression in HUVECs upon LPS stimulation. ISE pretreatment inhibited αvβ3 protein expression in a concentration-dependent manner (*n* = 5 independent experiments, Figure 5A–C). Correspondingly, ISE pretreatment inhibited the LPS-induced increase in endothelial permeability (Figure 2J–M) and vWF release from ECs (*n* = 5 independent experiments, Figure 3J,K). These findings suggest that ISE may prevent LPS-induced endothelial dysfunction by inhibiting the vWF-αvβ3 interaction. Thereafter, vWF’s concentration-dependent increase of endothelial permeability (*n* = 5 independent experiments, Figure 5D) and decrease of protein expression of VE-cadherin and occludin (*n* = 5 independent experiments, Figure 5E–G) were verified. The IC_50_ value of ISE protected against increased endothelial permeability induced by 100 ng/mL vWF was 37.5 μM (*n* = 5 independent experiments, Figure 5H). Endothelial permeability was significantly attenuated by pretreatment with 50 nM cilengitide or 50 μM ISE alone. However, the combination of 50 nM cilengitide and 50 μM ISE failed to enhance the inhibitory effect of ISE on endothelial injury compared to the 50 μM ISE pretreatment group (*n* = 5 independent experiments, Figure 5I–L).

Activation of the integrin αvβ3-FAK/Src signaling pathway is crucial for basic EC functions, such as cell adhesion and permeability [18]. VWF significantly increased both FAK (Y397) and Src (Try416) phosphorylation but not total protein expression levels. However, phosphorylated FAK and Src were concentration-dependently inhibited by ISE on vWF-induced HUVECs after 15 min of pretreatment (*n* = 5 independent experiments, Figure 5M–O). These results suggest that ISE can sustain the endothelial protective effects by blocking vWF-mediated integrin αVβ3-FAK/Src signaling pathways even after a 15 min pretreatment.

## 3. Discussion

Endothelial hyperpermeability and subsequent vascular leakage are hallmarks of sepsis, contributing to microcirculatory blood flow damage, tissue hypoperfusion, and life-threatening multiple organ failure. Despite its significance, effectively targeting endothelial hyperpermeability remains a challenging aspect of sepsis treatment. This study provides compelling experimental evidence supporting the protective effects of ISE against sepsis through regulation of endothelial hyperpermeability and platelet–endothelial interactions. The findings indicate ISE has direct effects on activated ECs by inhibiting vWF secretion and the interaction between vWF-integrin αvβ3 and downstream FAK/Src signaling. ISE also significantly reduced the increased release of vWF from activated platelets and platelet–EC interaction during sepsis.

According to the evidence, ISE, administered within a dose range between 25 mg/kg and 100 mg/kg, increases the survival rate and attenuates acute lung injury and systemic inflammation caused by CLP sepsis in a dose-dependent manner. Sepsis-induced platelet activation initiates microthrombus formation and rapid excessive platelet depletion, resulting in thrombocytopenia, a major cause of multiple organ failure [14,15]. Moreover, activated platelets secrete or generate agonists and cytokines, such as ADP, PF4, P-selectin, IL-1β, and vWF, which further amplify platelet activation and aggregation, fostering platelet–neutrophil interactions and NET formation, thereby fostering a proinflammatory and prothrombotic milieu during sepsis. Consequently, a reduced platelet count serves as a prognostic marker and predictor of poor outcomes in sepsis [16]. While antiplatelet and antithrombotic agents can improve survival and organ injury in animal models of sepsis, trials evaluating conventional antiplatelet drugs, such as P2Y_12_ receptor antagonists, have yet to establish their efficacy in reducing sepsis mortality. Thus, there remains a critical need for novel antiplatelet agents with alternative mechanisms of action [15]. Although our previous investigation revealed the antiplatelet and antithrombotic effects of ISE in a mouse model of FeCl_3_-induced carotid artery thrombosis [13], its effects in other thrombotic disease models remained unexplored. The present study found that ISE significantly attenuated CLP-induced sepsis by inhibiting pulmonary embolism and systemic inflammation and restoring depleted platelet counts, indicating its potential to protect against sepsis partly by mitigating platelet activation. The distinctive cyclodepsipeptides present in ISE may harbor novel antiplatelet molecular mechanisms and other undiscovered pharmacological effects against sepsis.

Notably, ISE pretreatment significantly ameliorated sepsis-induced pulmonary vascular permeability (Figure 2). During sepsis, enhanced pulmonary vascular permeability is mainly attributed to the disruption of endothelial barrier integrity. Syndecan-1 and endoglin are crucial components of the glycocalyx, the protective layer lining the luminal surface of ECs; their degradation contributes to microcirculatory dysfunction in sepsis [17]. VE-cadherin is expressed exclusively in ECs and maintains endothelial cell–cell junctions. Occludin is another critical transmembrane protein that is expressed at tight junctions in the endothelium. Both VE-cadherin and occludin expression are reduced in the lungs of CLP-induced septic mice [18]. Accumulating evidence shows that endothelial hyperpermeability is a key driver of sepsis pathology, and loss of endothelial barrier integrity contributes to vascular hyperpermeability development [3,19,20]. Experimental approaches targeting ECs specifically have yielded encouraging results. For instance, heparin (which protects against glycocalyx degradation), the Tie2 receptor agonist vasculotide (an angiopoietin-1 mimetic), and inhibitors of VE-cadherin internalization (via inhibition of the GTP-binding protein ARF6) are all promising therapies for reconstituting EC integrity during sepsis [3,21]. Moreover, ISE alleviated the disruption of pulmonary vascular permeability in septic mice, as evidenced by Evans blue dye extravasation assays and analysis of total protein and cells in bronchoalveolar lavage fluid. These findings support the hypothesis that ISE may prevent sepsis by acting directly on ECs. This was tested using LPS-stimulated ECs, which are a commonly used in vitro sepsis model. The results demonstrate the beneficial effects of ISE on LPS-induced disruption of endothelial permeability. This novel observation suggests that ISE acts directly on ECs, potentially contributing to its protective effects against sepsis-associated endothelial hyperpermeability.

vWF and vWF-mediated signaling pathways contribute to coagulation, platelet activation, adhesion, and endothelial activation, making them attractive therapeutic targets for various thrombotic and infectious diseases, including stroke, thrombotic thrombocytopenic purpura, sepsis, and COVID-19 [22,23,24]. Our previous research revealed that ISE specifically inhibits platelet activation and release with a relatively low IC_50_ value, suggesting that ISE affects vWF released from activated platelets during sepsis. Notably, the injured endothelium is another major source of elevated circulating vWF levels, especially under diseased conditions, such as vascular and infectious diseases [4,5,25,26,27]. For example, recent histological results in lung tissue from patients with COVID-19 have provided direct evidences showing clot formation with increased staining of EC-associated vWF [27]. Although a large amount of clinical evidence correlates the plasma levels of vWF with the severity and outcome of sepsis patients [28,29,30], there is no evidence of a correlation between local EC-vWF level with sepsis-induced lung injury. Here, we observed increased vWF in sepsis-induced lung sections, which was significantly reduced by ISE. Our results therefore supported the important role of EC-associated vWF in organ and tissue damage in many diseases. The significant inhibitory effect of ISE on EC-associated vWF levels may also provide an interesting direction for its application to other vascular and infectious diseases in the future.

High local concentrations of ULvWF strings can form net-like structures to capture platelets on injured ECs, resulting in increased platelet adhesion, aggregation, and initiation of the coagulation cascade. Given the high local concentration and unique network structure of vWF at vascular injury sites, drugs targeting vWF and vWF-mediated signaling pathways possess specific antithrombotic effects owing to their action at vascular injury sites, thereby reducing bleeding risk [31]. For instance, caplacizumab (ALX-0081), an anti-vWF A1 domain nanobody, is selectively used to treat acute thrombotic thrombocytopenic purpura by targeting the vWF-platelet glycoprotein 1bα interaction to inhibit microthrombosis formation [24]. In the present study, ISE reduced vWF release in LPS-stimulated HUVECs and inhibited platelet adhesion to LPS-stimulated HUVECs. Furthermore, the direct exposure of HUVECs caused impaired endothelial permeability, which was inhibited by ISE treatment. These findings suggest that the suppression of elevated vWF levels and increased platelet–EC interactions contribute to the protective effects of ISE against sepsis. Moreover, considering the emerging role of vWF as a selective antiplatelet target, the substantial inhibitory effect of ISE on elevated vWF levels resulting from activated platelets and injured endothelium might explain its significant antiplatelet effect and low bleeding risk.

Integrin αvβ3, a heterodimeric transmembrane cell adhesion molecule composed of αv- and β3-subunits, is prominently expressed on the surface of ECs [8]. It serves as a major vWF receptor on ECs [32] and is responsible for regulating endothelial permeability and angiogenesis [33,34]. Cilengitide, an integrin αvβ3 inhibitor, has been shown to inhibit the disruption of endothelial permeability induced by bacterial attachment [35]. This study revealed that ISE inhibited integrin αvβ3 expression in LPS-stimulated ECs. Notably, the combination of cilengitide and ISE produced comparable outcomes in mitigating vWF-mediated endothelial permeability disruption, underscoring the protective role of ISE against endothelial hyperpermeability during sepsis by suppressing the vWF–integrin αvβ3 interaction. The FAK/Src signaling pathway serves as the downstream mediator of integrin αvβ3 activation, regulating various biological processes, including angiogenesis [36,37]. Our findings demonstrate that vWF incubation significantly upregulated both phosphorylated FAK and Src protein expression, which was restored by ISE pretreatment without affecting their total protein expression. These results further reveal that ISE exerts its anti-sepsis effects by modulating the vWF/integrin αvβ3/FAK/Src signaling pathway in ECs. As our focus was primarily on exploring the effects of ISE on endothelial hyperpermeability, further investigation of other functional phenotypes of ECs will provide valuable insights into the pharmacological effects of ISE on sepsis-induced endothelial responses.

NETs interact with platelets and the endothelium to activate matrix metalloproteinases, thereby impairing endothelial integrity. Therapeutic approaches that reduce NET formation, such as peptidylarginase deaminase 4 and TLR4 inhibitors, can improve lung injury and attenuate inflammation in sepsis [38]. In this study, ISE pretreatment significantly decreased MPO plasma levels, a marker of NET burden induced by CLP-induced sepsis. This suggests that ISE improves sepsis by inhibiting NET-associated mechanisms.

Endothelial hyperpermeability is the main driver of multiple organ failure and is associated with high morbidity and mortality rates in patients with sepsis. Thus, clinical drugs and novel compounds with beneficial effects on sepsis-associated endothelial hyperpermeability are promising avenues for sepsis therapy. In this study, we demonstrated that ISE pretreatment significantly increased the survival rate of mice with CLP-induced sepsis and attenuated sepsis-induced lung injury. Maintenance of EC integrity, improved thrombocytopenia, decreased vWF levels, and vWF-mediated platelet-EC interactions are responsible for these anti-sepsis effects. The regulation of vWF binding to integrin αvβ3 and downstream FAK/Src signaling may explain the protective effects of ISE on endothelial hyperpermeability and warrant further investigation. The molecular mechanisms underlying the regulation of vWF release by ISE should also be investigated in future studies. Finally, given that ISE significantly improved sepsis-induced EC hyperpermeability and platelet activation with a low bleeding risk, it holds considerable potential for the development of new sepsis treatments.

## 4. Materials and Methods

### 4.1. Chemical Structure of Isaridin E

Isaridin E was obtained as a colorless crystal with a molecular formula of C_35_H_54_O_7_N_5_. The HR-ESIMS spectrum and NMR data of the molecule are described in our previous publication [13]. The chemical structure of ISE is depicted in Figure 1A. The HPLC data showed that the purity of ISE (*Rt* = 10.88 min) was >98.5% (Figure S1).

### 4.2. Preparations of ISE

For in vivo experiments, ISE was dissolved in a saline solution containing 10% Tween 80 and 15% propylene glycol (used as a vehicle control) at 2.5, 5, and 10 mg/mL [13]. For in vivo experiments, multiple intragastric administrations of ISE (25, 50, and 100 mg/kg/day) were applied at 48 h, 24 h, and 1 h before caecal ligation and puncture (CLP; Figure 1B). For in vitro experiments, ISE was dissolved in dimethyl sulfoxide (DMSO) and stored at 4 °C until use with a final concentration of DMSO < 0.1%.

### 4.3. Animals

The protocols for all experimental procedures were approved by the Sun Yat-sen University Animal Care and Use Committee (approval no. SYSU-IACUC-2020-B0112) and conducted in accordance with the “Guide for the Care and Use of Laboratory Animals” issued by the Ministry of Science and Technology of China.

Male C57BL/6 mice (6–8 weeks old, 21–23 g) were obtained from GemPharmatech, Nanjing, China, and housed under specific pathogen-free conditions. The CLP surgery was performed following established protocols [39]. Laparotomy was performed under anesthesia using 1.5% isoflurane (RWD, Shenzhen, China). Half the cecum was ligated and punctured twice with a 22-gauge needle, followed by resuscitation with an intraperitoneal injection of 1 mL of saline. Sham-operated mice underwent a similar laparotomy without ligation or double puncture. All mice were kept warm for 1 h post-surgery. Survival rates were monitored twice daily post-surgery, and mice were euthanized under anesthesia upon reaching the designed experimental endpoint. ISE pretreatment and CLP surgery were performed by different researchers to minimize the possible selection bias.

### 4.4. Hematoxylin and Eosin Staining

The lung samples were harvested and fixed in 4% paraformaldehyde (Sigma-Aldrich, St. Louis, MO, USA) for 48 h. Lung sections (5 μm) were stained with hematoxylin and eosin (H&E) following established procedures [40]. Pulmonary injury was evaluated using a lung injury grading system comprising four distinct categories: neutrophil infiltration, interstitial inflammation, oedema, and congestion. Five randomly selected tissue sections were used to calculate the average lung injury score, where scores assigned to four categories were averaged to acquire the overall lung injury score. The histological images were captured using Pannoramic MIDI (3DHISTECH, Budapest, Hungary).

### 4.5. Evans Blue Staining

Evans blue staining was employed to evaluate pulmonary vascular permeability [41]. Mice received an intravenous (*i.v.*) injection of 1% Evans blue dye (Sigma-Aldrich, St. Louis, MO, USA) diluted in normal saline. Lung tissues were harvested, and the dye was extracted by incubating the samples in 500 μL of formamide (Junsei, Tokyo, Japan) overnight at 60 °C. Subsequently, the samples were centrifuged at 14,000 rpm for 30 min, and Evans blue dye concentrations were determined by measuring absorbance at 630 nm.

### 4.6. Isolation and Culture of HUVECs

HUVECs were isolated and cultured following the protocol in our previous study [42]. The experimental protocol was approved by the Sun Yat-sen University Animal Care and Use Committee (No: SYSU-IACUC-2020-B0112). HUVECs were removed from human umbilical veins after collagenase type I digestion (Gibco, Grand Island, NY, USA) and cultured in 4 mL of ECM medium (Sciencell, Carlsbad, CA, USA) supplemented with 10% fetal bovine serum (Gibco, Grand Island, NY, USA) and 1% penicillin/streptomycin (Gibco, Grand Island, NY, USA) at 37 °C in 5% CO_2_. Passage 3–6 HUVECs were utilized for experiments.

### 4.7. Platelet Preparation

Washed platelets followed previously established methods [43]. Whole blood was centrifuged at 300× *g* for 10 min at room temperature to obtain platelet-rich plasma, which was then transferred to a fresh tube and centrifuged at 900× *g* for 5 min. The platelet pellet was resuspended in Tyrode’s buffer (134 mM NaCl, 12 mM NaHCO_3_, 2.9 mM KCl, 0.34 mM Na_2_HPO_4_, 1 mM MgCl_2_, 10 mM HEPES; pH 7.4) supplemented with 0.5 U/mL apyrase.

### 4.8. EC Permeability Assay

HUVECs were seeded at a density of 4 × 10^5^ cells/well onto 12-well transwell semipermeable supports (0.4 μm pore size; Corning Incorporated, Corning, NY, USA) coated with 1% gelatin. Following treatment, FITC-dextran (30 mg/mL; Sigma–Aldrich, St. Louis, MO, USA) was added to the upper chamber, and the cells were incubated for an additional 30 min. Absorbance was measured at 492 nm (excitation) and 520 nm (emission) using the Victor Nivo 5S (PerkinElmer, Waltham, MA, USA).

### 4.9. Platelet Adhesion Experiment in HUVECs

HUVECs in 24-well plates were incubated with ISE for 1 h, followed by stimulation with 10 ng/mL LPS or PBS for 6 h. Freshly isolated platelets were labelled with 6 μM CellTracker™ Green CMFDA Dye (Invitrogen, Carlsbad, CA, USA) at 37 °C for 30 min. The dye-labelled platelets were added to stimulated HUVECs at a ratio of 100:1, and the cells were further incubated at 37 °C for 1 h. Images were acquired at 20-times and 40-times magnifications under a Leica DMI8 inverted fluorescence microscope (Leica, Wetzlar, Germany).

### 4.10. Measurement of Cytokine Levels

Elisa assays were performed following established protocols [44]. Briefly, ELISA kits for PF4, IL-1β, TNF-α, MPO (mibio, Shanghai, China), endoglin (MULTISCIENCES, Hangzhou, China), vWF, syndecan, and ADAMTS-13 (Cusabio, Wuhan, China) were used according to the manufacturer’s protocol. Optical absorbance was measured at 450 nm using a Sunrise^TM^ ELISA plate reader (Tecan, Zurich, Switzerland). Standard curves were generated by plotting the average optical density of the standard samples along the Y-axis against the corresponding concentration on the X-axis. Sample concentrations were performed with GraphPad Prism (Version 9.5.1, La Jolla, CA, USA).

### 4.11. Western Blot Analysis

For Western blot analysis, conventional or low-temperature SDS/PAGE were employed, as previously outlined [43]. Samples were separated on 8% SDS polyacrylamide gels and transferred onto nitrocellulose membranes. After incubation with primary antibodies, the membranes were kept at 4 °C overnight. Following this, nitrocellulose membranes were exposed to corresponding secondary antibodies conjugated with horseradish peroxidase (HRP) for 1.5 h at room temperature. Chemiluminescent signals emitted by protein bands were detected using an enhanced chemiluminescence (ECL) detection system (Millipore), and images were captured using an imaging system (Bio-Rad, Hercules, CA, USA). Optical densities were normalized to those of β-actin, and the fold difference for each target protein was calculated as the ratio of the target protein expression to β-actin expression using the ImageJ 2.1.0 software from the U.S. National Institutes of Health.

### 4.12. Statistical Analysis

The data are expressed as means ± SEM. All statistical analyses were performed using GraphPad Prism (Version 9.5.1, GraphPad Software, La Jolla, CA, USA). Student’s *t* test was used to compare two groups. Three or more groups were compared using one-way analysis of variance, followed by Dunnett’s multiple comparisons post-hoc test with a 95% confidence interval (CI). A *p* value of <0.05 was considered statistically significant.

## Data Availability

All data included in this study are available upon request by contact with the corresponding author.

## Supplementary Materials

The following supporting information can be downloaded at: https://www.mdpi.com/article/10.3390/md22060283/s1, Figure S1: The HPLC diagram of sample of isaridin E. The analysis was performed on an Agilent ZORBAX Eclipse Plus C18 column (3.5 μm, 4.5 × 100 mm) using a linear gradient of MeCN and water (0−2 min: 30% MeCN, 2–15 min: 30% MeCN–100% MeCN, 15–25 min 100% MeCN, 25–26 min: 100% MeCN-30% MeCN, 26–30 min 30% MeCN, 0.8 mL/min; 210 nm; 30 °C; 10 μL).
