# Supplementary material for: Isaridin E Protects against Sepsis by Inhibiting Von Willebrand Factor-Induced Endothelial Hyperpermeability and Platelet–Endothelium Interaction
Source: Mar Drugs. 2024 Jun 16;22(6):283. doi: 10.3390/md22060283 (PMC11204489; doi:10.3390/md22060283)
Supplement: Supplementary file 1 [file marinedrugs-22-00283-s001.zip › marinedrugs-3005875-supplementary.pdf]

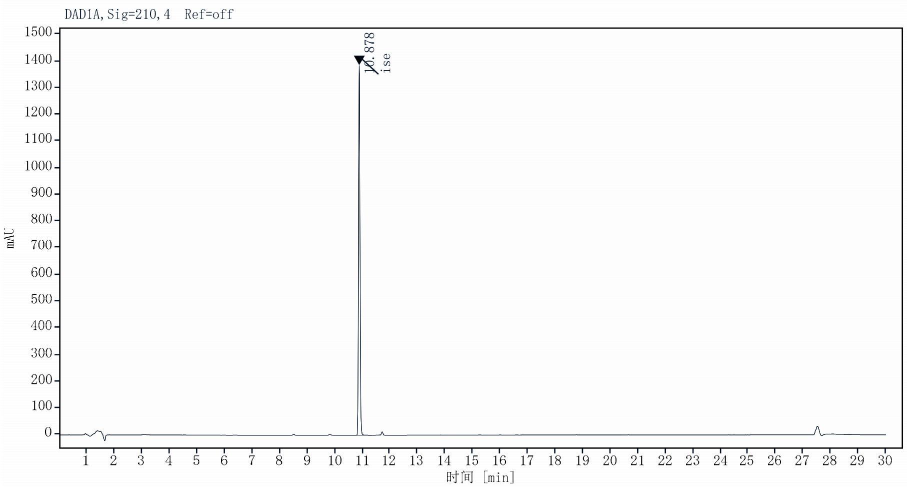

Figure S1. The HPLC diagram of sample of isaridin E. The analysis was performed on an Agilent ZORBAX Eclipse Plus C18 column (3.5 μm, 4.5×100 mm) using a linear gradient of MeCN and water (0−2 min: 30% MeCN, 2-15 min: 30% MeCN − 100% MeCN, 15-25 min 100% MeCN, 25-26 min: 100% MeCN-30% MeCN, 26-30 min 30% MeCN, 0.8 mL/min; 210 nm; 30°C; 10 μL).
